# Association of Malocclusion with Temporomandibular Disorders: A Cross-Sectional Study

**DOI:** 10.3390/jcm13164909

**Published:** 2024-08-20

**Authors:** David Faustino Ângelo, Maria Cristina Faria-Teixeira, Francesco Maffia, David Sanz, Marcella Sarkis, Rute Marques, Beatriz Mota, Ricardo São João, Henrique José Cardoso

**Affiliations:** 1 Instituto Português da Face, 1500-493 Lisbon, Portugal; francesco.maffia@gmail.com (F.M.); 27168@chln.min-saude.pt (B.M.); 2 Centre for Rapid and Sustainable Product Development, Polytechnic Institute of Leiria, 2430-028 Marinha Grande, Portugal; 3 Faculty of Medicine, Lisboa University, 1649-028 Lisbon, Portugal; 4 Serviço de Estomatologia Hospital Egas Moniz, Centro Hospitalar de Lisboa Ocidental, 1349-019 Lisbon, Portugal; 5 Centro Hospitalar Universitário Lisboa Norte (CHUNL), Clinica Universitária de Estomatologia, 1648-028 Lisbon, Portugal; 6 Maxillofacial Surgery Unit, Department of Neurosciences, Reproductive and Odontostomatological Sciences, University of Naples “Federico II”, Via Sergio Pansini 5, 80131 Naples, Italy; 7 Department of Computer Science and Quantitative Methods, School of Management and Technology, Polytechnic Institute of Santarém, 2001-904 Santarém, Portugal; 8 CEAUL—Centro de Estatística e Aplicações, Faculdade de Ciências, Universidade de Lisboa, 1749-016 Lisbon, Portugal; 9 Center for Global Studies (CEG-UAb), Aberta University, 1250-100 Lisbon, Portugal; 10 Nursing Research, Innovation and Development Center of Lisbon (CIDNUR), 1600-190 Lisbon, Portugal

**Keywords:** temporomandibular disorders, dental occlusion, bruxism, malocclusion

## Abstract

**Background/Objectives**: Temporomandibular disorders (TMD) encompass a range of musculoskeletal and neuromuscular conditions affecting the temporomandibular joint (TMJ) and associated structures. This cross-sectional study, conducted in a Portuguese TMD department, aimed to assess the relationship between malocclusion and TMD severity. **Methods**: Data on demographic variables, TMD clinical symptoms, and malocclusion classes were collected using the EUROTMJ database. The Chi-square test (χ^2^) identified associations, with their intensity measured by Cramér’s V (φc). **Results**: The study included 1170 patients (932 females and 238 males), with a mean age of 41.73 ± 16.80 years. Most patients exhibited Angle Class I malocclusion (85.5%), followed by Angle Class II (13.5%) and Angle Class III (1.1%). Class II malocclusion was associated with increased TMD severity (*p* < 0.001), higher myalgia levels (*p* = 0.002), more frequent disc displacement without reduction (*p* = 0.002) and lower maximum mouth opening values (Class II: 38.13 ± 7.78 mm, Class I: 39.93 ± 8.67 mm). Significant associations were also found between malocclusion type and arthralgia (*p* = 0.021), mouth-opening limitation (*p* = 0.016), and TMJ crepitus (*p* = 0.017). In cases of malocclusion, the presence of oral signs of bruxism explained the degree of myalgia, disc displacement, and severity (*p* = 0.003; *p* = 0.048; *p* = 0.045). **Conclusions**: This study highlights that (1) the most common type of dental malocclusion in TMD patients was Class I; (2) Class II malocclusion was associated with increased TMD severity and oral signs of bruxism; and (3) Class III was rarely observed in TMD consultation. The findings suggest that bruxism behavior in cases of malocclusion may be significant in TMD.

## 1. Introduction

Temporomandibular disorders (TMD) are a group of musculoskeletal and neuromuscular conditions involving the temporomandibular joint (TMJ), masticatory muscles, and all associated structures [1,2]. TMD include abnormalities in the intra-articular disc position and/or dysfunction of the related musculature [3,4]. Common signs and symptoms are TMJ pain, clicking, masticatory muscle tension, headache, and restricted mouth opening [5]. Notably, TMD symptoms disproportionately affect females, with reported female-to-male ratios ranging from 2:1 to 8:1 [4,6,7]. 

A significant subset of TMD patients—up to 70%—suffers from internal derangement (ID) of the TMJ disc. Despite its prevalence, TMD progression is poorly understood and often associated with degenerative conditions such as osteoarthritis (OA) [1,8]. Thorough clinical evaluation, including physical examinations and imaging, is crucial for accurate diagnosis. Various questionnaires and indexes help to describe and validate TMD diagnoses and symptom severity [1,8]. The primary clinical management goals are to increase the mandibular range of motion, decrease joint and muscle pain, and prevent further degenerative changes. 

TMD affect 25–38% of the general population [9,10] and are influenced by factors like maxillofacial traumas, parafunctional habits, functional overloading, increased joint friction, metabolic conditions, unstable occlusion, orthodontic treatment, wisdom teeth removal, sleep and psychosocial factors [4,11]. The role of these factors remains controversial due to inconsistent evidence of cause-and-effect relationships. Potential confounding factors, such as age, sex, familiarity, or environment, also play significant roles [12,13]. 

The link between malocclusion and TMD is particularly contentious. An unstable occlusion might disrupt TMJ load-bearing capacity, possibly contributing to TMD development [14]. While tooth-clenching and grinding are acknowledged as significant factors, the exact contribution of malocclusion remains debated. 

This research aims to fill the gap in understanding the role of malocclusion in TMD, providing new insights and highlighting limitations in existing research. Our objectives were to assess (1) the type of malocclusion of TMD patients seeking specialist appointments and (2) the severity of TMD symptoms and their possible association with the type of malocclusion. 

## 2. Materials and Methods

### 2.1. Study Design 

This cross-sectional study was conducted over five years at a Portuguese TMD department, from 1 January 2019 to 1 January 2024. This study was approved by the Instituto Português da Face Ethics committee (reference number: PT/IPFace//RCT/0119/2402). Participants provided voluntary written consent after being informed about the study’s purpose, procedures, risks, and benefits, with assurances of confidentiality according to current legislation. 

### 2.2. Data Collection

The inclusion criteria included (1) comprehensive documentation of all relevant variables during the initial consultation and (2) a clinical and imaging diagnosis of unilateral or bilateral TMD. Exclusion criteria consisted of the following: (1) severe medical conditions or evidence of impaired cognitive capacity, (2) patients with tooth loss, and (3) any history of previous TMJ surgical intervention or any facial trauma within the previous 4 weeks before the study. 

### 2.3. Variables Recorded

Data were recorded in the European Temporomandibular Joint (EUROTMJ) database, including demographic data, Angle’s occlusion classification, mouth-opening limitation, maximum mouth-opening (MMO), the presence of clicks, crepitus, myalgia, and arthralgia. Bruxism signs were also registered during intra-oral examinations. This database’s functioning and registration variables have already been previously described [4]. 

Dental malocclusion was assessed during the clinical examination and registered according to Angle’s occlusion classification based on the relationship between the mesiobuccal cusp of the maxillary first molar and the buccal groove of the mandibular first molar (Class I malocclusion, Class II malocclusion, Class III malocclusion).

TMD symptoms were assessed using several specific tools: the Diagnostic Criteria for Temporomandibular Disorders (DC/TMD), the Visual Analog Scale (VAS), and the TheraBite Jaw range-of.motion (ROM) Scale. 

Mouth opening limitation was defined as encountered when (1) patients perceived a limitation in their mouth opening; (2) it was clinically observed; and (3) the measured MMO was <40 mm [15,16]. MMO was assessed using a certified ruler between the upper and lower incisors (TheraBite Jaw ROM Scale). 

Myalgia was diagnosed based on a history of jaw pain in the past 30 days, pain in front of or directly in the ear, and clinical confirmation through palpation of the masticatory muscles. The palpation pressure was standardized (5 s/1 kg pressure) in the masseter and temporalis muscles, following the DC/TMD guidelines. [15,16]. Myalgia was graded according to pain severity: 0 = No Pain/Pressure Only; 1 = Mild Pain; 2 = Moderate Pain; and 3 = Severe Pain [17]. 

Crepitus and clicks were clinically assessed and registered when present during mouth-opening and mouth-closing movements.

Arthralgia was reported if verified if (1) there was a history of pain in the TMJ area, and (2) it was accompanied by jaw movement, function, or parafunction [15,16]. The level of TMJ arthralgia was registered through the VAS (0–10, with 0 being no pain and 10 being severe pain), assessing the pain on palpation of the lateral pole area or registering the pain during maximum unassisted/assisted opening or lateral or protrusive movements. 

The position of the articular disc was determined using magnetic resonance imaging (MRI). It was classified as follows: absence of displacement (woD), displacement of the disc with reduction (DDwR), displacement of the disc without reduction (DDwoR), or the presence of one side with reduction and the other without reduction (DDwR/DDwoR). 

The severity of TMD was assessed according to the Dimitroulis classification: category 1—normal TMJ; category 2—minor TMJ changes; category 3—moderate TMJ changes; category 4—severe TMJ changes; and category 5—catastrophic TMJ changes [18]. 

The same clinician (D.F.A) carried out all clinical assessments and diagnoses. Inter-rater reliability was ensured by standardizing training and assessment protocols for all clinicians and conducting regular calibration meetings. Consistency was monitored through periodic comparisons of evaluations between different raters to maintain high agreement levels.

### 2.4. Statistical Analysis 

A power analysis was performed to achieve a statistical power of 80% with a significance level of 5%. Data were analyzed using IBM SPSS (v26, Armonk, NY, USA) software. 

The data were analyzed using IBM SPSS (v26, Armonk, NY, USA). Non-parametric tests, including the Chi-square test (χ^2^) and Mann–Whitney test, were chosen because the data did not meet the assumptions required for parametric tests, such as normal distribution. The Chi-square test assessed associations between categorical variables, while the Mann–Whitney test compared medians between groups. Cramér’s V Coefficient (φc) was used to measure the strength of associations. A *p*-value of <0.05 was considered statistically significant.

## 3. Results

A total of 1170 patients were registered in the EUROTMJ database with a mean age of 41.73 ± 16.80 years. Nine hundred thirty-two patients were females, representing 79.7% of the sample. 

Clinical evaluation revealed that 683 patients (63.7%) had arthralgia, with a VAS level of 6.54 ± 2.54. Mouth opening limitation was observed in 684 patients (58.5%), with an MMO of 38.59 ± 8.46 mm (Table 1). The mean myalgia degree was 2.16 ± 1.04. TMJ crepitus and clicks were present in 251 (23.3%) and 541 (50.2%) patients, respectively (Table 1). Disc displacement with reduction (DDwR) was noted in 396 patients (33.8%), while 265 (22.6%) had disc displacement without reduction (DDwoR), and 87 patients presented one side with DDwoR and other with DDwR (7.4%). In 299 patients (25.6%), no disc displacement was observed. Regarding the Dimitroulis Classification, 317 patients (27.3%) were included in Category 1 (Normal TMJ), 413 patients (35.6%) in Category 2 (TMJ minor changes), 228 in Category 3 (19.7%) (TMJ moderate changes), 196 patients (16.9%) in Category 4 (TMJ severe changes) and 6 (0.5%) in Category 5 (Catastrophic TMJ) (Table 1). Oral signs of bruxism were observed in 368 patients (31.5%).

**Figure 1:**
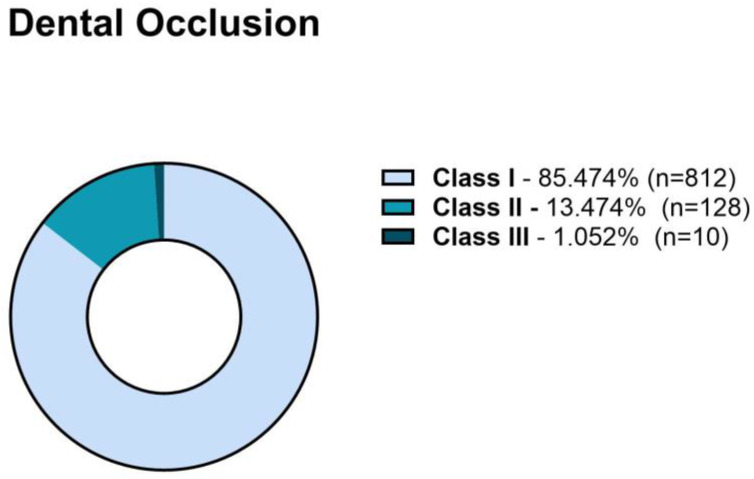
Malocclusion Angle classification of the patients who attended a consultation for temporomandibular disorders (TMD).

Dental malocclusion was assessed in 950 patients. Angle’s classification revealed that 812 patients (85.474%) had Class I malocclusion, 128 patients (13.474%) had Class II malocclusion, and 10 (1.05%) patients had Class III malocclusion (Figure 1). 

This article aimed to assess whether there was any association between the TMD clinical variables and the type of malocclusion. Class III malocclusion showed a low causality for statistical comparison purposes. Thus, only Class I and II comparisons were considered (Table 2). 

While assessing the association of clinical variables with the type of malocclusion, we observed that arthralgia, limitation of mouth opening, and TMJ crepitus had an association (*p* = 0.021; *p* = 0.016; *p* = 0.017, Table 2). All associations were weak according to Cramér’s V Coefficient (φc = 0.075; φc = 0.078; φc = 0.078). It should be noted that there was a higher percentage of Class II patients with arthralgia and limited mouth opening and crepitus when compared to Class I patients (73.0% vs. 62.8%; 69.6% vs. 58.7%; 30.7% vs. 21.4%, respectively, Table 2). 

Oral signs of bruxism also presented a weak association with the type of malocclusion (*p* = 0.043, φc = 0.066, Table 2). Seventy-six percent (75.7%) of Class II patients showed oral signs of bruxism, compared with 67.0% of Class I patients.

Myalgia degree and disc position were also associated with the type of malocclusion (*p* = 0.002); *p* = 0.002, Table 2). Both associations were moderate (φc = 0.104; φc = 0.0128). Class II patients had the highest levels of myalgia, with 87.7% exhibiting a myalgia degree from 2 to 3. In addition, Class II patients had a higher association with DDwoR than Class I patients (36.6% vs. 24.2%, Table 2). Class II patients also had significantly lower MMO values than Class I patients (38.13 ± 7.78 vs. 39.93 ± 8.67; *p* = 0.01, Z = −2.575, Table 2). Class III patients had higher MMO averages than Classes I and II (42.40 ± 5.85 vs. 38.13 ± 7.78 and 39.93 ± 8.67, Table 2). 

**Figure 2:**
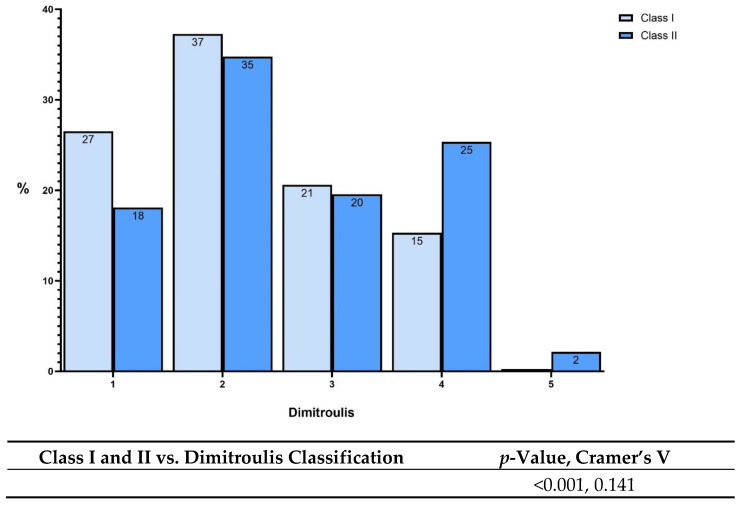
Distribution of patients with malocclusion I and II by the different degrees of severity using the Dimitroulis classification.

We further assessed whether there was an association between the malocclusion class and the TMD severity assessed by the Dimitroulis classification (Figure 2). An association was identified between the malocclusion type and the TMD severity (*p* < 0.001, Figure 2). The association was moderate (φc = 0.141). Figure 2 shows that Class II patients had increased TMD severity compared to Class I patients (Dimitroulis category 4— 25% vs. 15%, Figure 2). In line with this, Class I patients had a higher percentage of patients in low severity categories when compared to Class II patients (Dimitroulis category 1—27% vs. 18%, Figure 2).

Next, we checked whether signs of bruxism could be associated with the results obtained in cases of malocclusion (Table 3). It was found that in cases of malocclusion, patients with bruxism were associated with higher levels of myalgia, more cases of DDwoR, and a higher degree of severity (*p* = 0.003; *p* = 0.048; *p* = 0.045, Table 2). This association was strongly related (φc = 0.319; φc = 0.240; φc = 0.267). 

Finally, we checked for associations between previous orthognathic surgery, orthodontic treatment, and the Class of malocclusion. No associations were found (Table 4).

## 4. Discussion

This study aimed to explore the relationship between malocclusion classes and TMD severity, focusing on the association between malocclusion patterns and clinical manifestations such as myalgia, disc displacement, and limited mouth opening. Our findings contribute to the existing body of research on TMD by highlighting specific craniofacial features and malocclusion patterns that may influence TMD severity, particularly in Class II malocclusion cases. 

Our results show that Class I malocclusion was the most prevalent among TMD patients, but Class II malocclusion was linked to more severe symptoms. Patients with Class II malocclusion exhibited higher rates of arthralgia, myalgia, and restricted mouth opening compared to those with Class I. Although no statistical test was carried out, it is essential to note that Class III appears to be associated with some variables, namely high levels of myalgia, limited mouth opening, and disc displacement. Studying this Class of malocclusion with a larger sample in the future would be necessary. Notably, the association between TMD severity and Class II malocclusion was further supported by the Dimitroulis classification, which revealed that Class II patients had a higher percentage of severe cases. Recent craniofacial studies assessing dental malocclusion and TMD symptoms report that patients presenting Class I malocclusion frequently revealed no signs of TMD. At the same time, 70% of Class II individuals had TMD-related mild symptoms, according to the Fonseca Anamnestic Index [13]. 

Muscular variables play an essential role in stomatognathic balance and TMJ stability. Recent studies show that the lateral pterygoid muscle (LPM) plays a central role in disc and condyle rotation [19]. Morphological changes in the LPM observed in Class II patients could be related to internal derangement and abnormal disc movements [19,20]. Several studies have shown an association between anterior disc displacement and hyperdivergent Class II malocclusion [21,22,23,24,25,26,27,28]. Investigations [29,30] suggest an etiologic role of TMJ internal derangement in the dysmorphic development of the craniofacial complex and consequent malocclusion, based on the concept that the condyle represents a crucial growth center. Disc displacement can lead to compressive stress, decreased lubrication surfaces, inflammation, and tissue damage, resulting in pain, myalgia, and condylar and ramus height reduction, classically observed in Class II patients [14].

Class II patients also had significantly lower MMO values than Class I patients. Class III patients had higher MMO averages than Classes I and II patients. The literature suggests that Class II patients typically exhibit reduced condylar dimensions compared with those with Class III, but their glenoid fossae volumes are similar [25,31]. Moreover, the condyle/fossa matching degree correlates negatively with positional variation. More prominent condyles and similar glenoid fossae in Class III patients are related to a better condyle/fossa matching degree and less condylar displacement [25,31]. Therefore, the condyle/fossa matching degree and articular stability in patients with Class II seem significantly smaller than in patients with skeletal Class III (SCIII). We hypothesize that the condyle/fossa matching degree could contribute to the increased mouth opening length observed in Class III patients. 

Morphometric studies suggest that particular craniofacial features are often found in severe skeletal Class II patients. These features include mandibular retrusion, clockwise rotation of the mandible, smaller ramus height, decreased posterior facial height, larger occlusal angle, and a smaller sagittal position of the mandible (SNB). The same skeletal features are also related positively to the progression of degenerative joint disease or TMD signs and symptoms in adults [14,32,33]. Additionally, degenerative joint disorders are linked to a mandibular ramus deficiency, a larger gonial angle, a clockwise rotation of the mandible, a retrognathic appearance, and a vertically elongated facial pattern [14]. These features lead to a skeletal Class II malocclusion.

In this study, a higher percentage of Class II patients showed oral signs of bruxism compared to Class I patients. Among the patients with malocclusion who exhibited oral signs of bruxism, there was a higher degree of myalgia, more cases with DDwoR, and more cases of category 4 according to the Dimitroulis Classification. This aligns with the literature suggesting that bruxism, particularly clenching, may increase joint loading and muscle tension, contributing to the development or exacerbation of TMD symptoms. Recent research has provided preliminary support for this concept [34,35]. The correlation between bruxism and malocclusion needs further exploration in future studies. 

Finally, we investigated possible associations between previous orthognathic surgery, orthodontic treatment, and class of malocclusion. No associations were found. Despite previous reports on the effect, the impact of orthognathic surgery on the signs and symptoms of TMD is still controversial [36]. The recent literature suggests orthognathic surgeries could positively affect TMD, reflecting the correlation between skeletal malocclusion and TMD [37,38,39]. One particular study reported a decrease in TMD symptoms prevalence after orthognathic surgery, although the overall positive effect was less effective in skeletal Class II patients [40]. Some patients experienced postoperative condylar resorption, with those having preexisting TMJ dysfunction more likely to have worsening TMD after orthognathic surgery, especially in skeletal Class II patients [41]. Thus, malocclusion patterns seem to significantly influence the skeletal and muscular transmission of occlusal force in the stomatognathic system, affecting the adaptive remodeling of the TMJ [42]. 

Regarding the association between orthodontic treatment and TMD, evidence shows that orthodontic treatment can potentially influence TMD, but the exact nature of this relationship is complex and not fully understood [43,44,45]. While some studies suggest that orthodontic interventions might alleviate or exacerbate TMD symptoms, the evidence remains mixed [46]. Recent research with Class II and Class III patients has reported that the use of intermaxillary elastics increased the strain in the TMJ, particularly among Class II patients [47]. In contemporary orthodontics, adult patients frequently choose transparent aligners for their orthodontic treatment. Recent research shows an increased electromyographic activity of the masticatory muscles after 6 months of treatment with transparent aligners [48]. However, the potential relationship between the increased strain and the development of TMD signs and symptoms remains unknown and needs investigation in future studies [47]. 

The potential association between malocclusion and TMJ dysfunction remains a subject of debate. The current literature shows that several occlusal factors may contribute to developing signs and symptoms of TMD, with a higher prevalence of TMD symptoms in patients with dentofacial deformities and consequent malocclusion [49]. The potential association between malocclusion and TMJ disorders suggests that TMD arise as a malocclusion pattern may create an imbalance between the load exerted on the TMJs and their capability of bearing the load [14]. Most of the current literature questions the causal role of occlusion and occlusal disharmony in TMD etiopathogenesis [50]. In a systematic review, Lekaviciute, et al. [51] concluded that there is insufficient evidence to establish a direct and consistent relationship between occlusal class and TMD. They highlight that, although some studies suggest a possible association, the evidence is not robust enough to confirm a clear link [51]. Instead, the relationship between occlusal factors and TMD is considered complex and multifaceted, involving a combination of biomechanical, neuromuscular, and psychosocial factors. Our study contributes to this discussion by providing empirical evidence of the correlation between specific malocclusion types and TMD symptoms. However, it is essential to acknowledge the complexity of TMD etiology, which involves multifactorial influences beyond occlusal factors alone. The findings emphasize the need for a comprehensive diagnostic approach that considers both occlusal and non-occlusal factors in managing TMD.

As a cross-sectional study, our research is limited in establishing causality between malocclusion and TMD severity. The findings reflect associations rather than cause-and-effect relationships, which would require longitudinal studies to confirm. Additionally, our study did not include a control group of individuals without TMD, limiting our ability to compare the prevalence of malocclusion patterns in the general population. Other limitations include potential biases related to the single-center design and the lack of morphological or cephalometric assessments. Moreover, we did not include patients with tooth loss, which could influence occlusal patterns and subsequently affect TMD symptoms [51,52]. 

The clinical implications of our findings are that oral surgeons should consider occlusal factors when diagnosing and treating TMD. Recognizing the potential impact of Class II malocclusion on TMD severity can aid in developing more targeted and effective treatment strategies. Future research should focus on longitudinal studies to better understand the causative relationships and incorporate comprehensive imaging and morphological assessments to provide a more nuanced understanding of the interplay between malocclusion and TMJ function.

## 5. Conclusions

This study indicates that Class II malocclusion is associated with increased severity of TMD symptoms, including higher levels of myalgia and more frequent cases of disc displacement without reduction. While Class I malocclusion was the most prevalent in our sample, Class II patients exhibited more severe symptoms, highlighting the potential influence of specific craniofacial features on TMD. These conclusions are drawn considering the significant limitations of our study, including its cross-sectional nature and lack of a control group. The results underscore the importance of occlusal factors in TMD diagnosis and treatment. Future research should aim to confirm these findings through longitudinal studies and further explore the underlying mechanisms. This study contributes valuable insights into the complex relationship between malocclusion patterns and TMD, emphasizing the need for a comprehensive approach to patient care and management.

## Figures and Tables

**Table 1:** Global clinical outcomes. DDwR—disc displacement with reduction; DDwoR—disc displacement without reduction; DDwR/DDwoR—disc displacement with reduction and without reduction; MMO—maximum mouth opening; SD—standard deviation; TMJ—temporomandibular joint; woD—absence of displacement.

Clinical Outcome	Mean ± SD or *n* (%)
Arthralgia	683 (63.7%)
TMJ arthralgia level (VAS, 0–10)	6.54 ± 2.54
Mouth opening limitation	684 (58.5%)
MMO (mm)	39.59 ± 8.46
Myalgia Degree (0–3)	2.16 ± 1.04
TMJ Crepitus	251 (23.3%)
TMJ Clicks	541 (50.2%)
Disc Position	
woD	299 (25.6%)
DDwR	396 (33.8%)
DDwoR	265 (22.6%)
DDwR/DDwoR	87 (7.4%)
Dimitroulis Classification	
1	317 (27.3%)
2	413 (35.6%)
3	228 (19.7%)
4	196 (16.9%)
5	6 (0.5%)
Oral Signs of Bruxism	368 (31.5%)

**Table 2:** Clinical outcomes comparing dental malocclusion Class I vs. Class II vs. Class III. DDwR—disc displacement with reduction; DDwoR—disc displacement without reduction; DDwR/DDwoR—disc displacement with reduction and without reduction; MMO—maximum mouth opening; SD—standard deviation; TMJ—temporomandibular joint; woD—absence of displacement.

Clinical Outcome	Class I Mean ± SD or *n* (%)	Class II Mean ± SD or *n* (%)	Class III Mean ± SD or *n* (%)	Class I vs. II *p*-Value, Cramer’s V or U, *z*
Arthralgia	506 (62.8%)	100 (73.0%)	6 (60.0%)	0.021, 0.075
TMJ arthralgia level (VAS, 0–10)	6.62 ± 2.50	6.57 ± 2.45	6.56 ± 2.53	0.749, 48,120, −0.319
Limitation of mouth opening	477 (58.7%)	96 (69.6%)	7 (70.0%)	0.016, 0.078
MMO (mm)	39.93 ± 8.67	38.13 ± 7.78	42.40 ± 5.85	0.01, 63,060, 5, −2.575
Myalgia Degree				0.002, 0.104
0	114 (14.1%)	12 (8.7%)	1 (10.0%)	
1	74 (9.1%)	7 (3.6%)	0 (0%)	
2	216 (26.7%)	49 (35.5%)	5 (50.0%)	
3	406 (50.1%)	72 (52.2%)	4 (40.0%)	
TMJ clicks	405 (50.0%)	66 (47.8%)	2 (20.0%)	0.637, −0.015
TMJ crepitus	173 (21.4%)	42 (30.7%)	1 (10.0%)	0.017, 0.078
Oral signs of bruxism	534 (67.0%)	103 (75.7%)	6 (60.0%)	0.043, 0.066
Disc Position				
woD	226 (29.4%)	33 (24.6%)	2 (22.2%)	0.002, 0128
DDwR	305 (39.6%)	37 (27.6%)	1 (11.1%)	
DDwoR	186 (24.2%)	49 (36.6%)	5 (55.6%)	
DDwR/DDwoR	53 (6.9%)	15 (11.2%)	1 (11.1%)	

**Table 3:** Clinical outcomes compared in cases of malocclusion and comparing patients with/without oral signs of bruxism. DDwR—disc displacement with reduction; DDwoR—disc displacement without reduction; DDwR/DDwoR—disc displacement with reduction and without reduction; MMO—maximum mouth opening; SD—standard deviation; TMJ—temporomandibular joint; woD—absence of displacement.

Clinical Outcome	Without Oral Signs of Bruxism Mean ± SD or *n* (%)	Oral Signs of Bruxism Mean ± SD or *n* (%)	Class I vs. II *p*-Value, Cramer’s V or U, *z*
Arthralgia	21 (61.8%)	78 (76.5%)	0.095, 0.143
TMJ arthralgia level (VAS, 0–10)	5.76 ± 2.88	6.75 ± 2.30	0.131, 1390, 5, −1.51
Limitation mouth opening	23 (67.6%)	72 (69.6%)	0.805, 0.021
MMO (mm)	39.50 ± 9.62	37.99 ± 6.98	0.090, 1411, 5, −1.69
Myalgia Degree			0.003, 0.319
0	7 (20.6%)	4 (4.9%)	
1	3 (8.8%)	2 (1.9%)	
2	13 (38.2%)	36 (35.0%)	
3	11 (32.4%)	60 (58.3%)	
TMJ clicks	18 (52.9%)	48 (46.6%)	0.521, −0.055
TMJ crepitus	8 (23.5%)	34 (33.3%)	0.284, 0.092
Disc Position			
woD	10 (30.3%)	23 (23.0%)	0.048, 0.240
DDwR	14 (42.4%)	23 (23.0%)	
DDwoR	7 (21.2%)	41 (41.0%)	
DDwR/DDwoR	2 (6.1%)	13 (13.0%)	
Dimitroulis Classification			
1	7 (20.6%)	16 (15.5%)	
2	15 (44.1%)	32 (31.1%)	
3	9 (26.5%)	19 (18.4%)	0.045, 0.267
4	2 (5.9%)	34 (33.0%)	
5	1 (2.9%)	2 (1.9%)	

**Table 4:** Comparing dental malocclusion Class I vs. Class II according to previous treatments.

Previous Treatment/Risk Factor	Class I *n* (%)	Class II *n* (%)	*p*-Value, Cramer’s V
Orthognathic surgery	1 (0.1%)	0 (0.0%)	0.680, 0.013
Orthodontic treatment	117 (14.4%)	25 (18.1%)	0.259, 0.037

## Data Availability

The data presented in this study are available on request from the corresponding author.
